# Chelerythrine Protects Against Acetaminophen-Induced Acute Liver Injury: Insights from Gut Microbiota and Multi-Omics Analysis

**DOI:** 10.3390/antiox14091063

**Published:** 2025-08-29

**Authors:** Jinlong Liu, Yanfei Zhang, Hao Wu, Pan Yang, Wenlong Wang, Chenliang Li, Hong Cao, Jinying Wu, Xin Sun

**Affiliations:** School of Pharmaceutical Sciences, Jilin Medical University, No.5, Jilin Street, Fengman District, Jilin 132013, China; jinlongliu@jlmu.edu.cn (J.L.); zyf20181812@163.com (Y.Z.); haowu@jlmu.edu.cn (H.W.); 15699571396@163.com (P.Y.); 13944674691@163.com (W.W.); lcl07292025@163.com (C.L.); 13234351232@163.com (H.C.); 13944628227@163.com (J.W.)

**Keywords:** chelerythrine, acute liver injury, *Barnesiella intestinihominis*, multi-omics

## Abstract

Chelerythrine (CHE) is the main active component of *Chelidonium majus* L., possessing excellent antioxidant and anti-inflammatory properties. However, the protective effects of CHE against liver injury and its underlying mechanisms remain unclear. We aimed to investigate the effects of CHE on acute liver injury (ALI) and explore its underlying mechanisms. Mice were orally administered with or without CHE (15 and 30 mg/kg) treatment for 7 days, followed by a single intraperitoneal injection of acetaminophen (APAP, 350 mg/kg). After 24 h, serum, liver, and fecal samples were collected. Then, 16S rRNA gene sequencing, metabolomics, and transcriptomics approaches were employed to investigate the protective effects of CHE against ALI. Finally, we elucidated the role of CHE in restoring gut microbiota and metabolic disorders in the context of ALI. The results showed that CHE significantly inhibited ALT and AST levels (*p* < 0.001). Furthermore, CHE counteracted APAP-induced alterations in IL-6, IL-1β, TNF-α, MPO, MDA, H_2_O_2_, CAT, SOD, and GSH (*p* < 0.05). These results indicate that CHE possesses antioxidant properties and inhibits inflammatory factors, thereby protecting the organism from APAP-induced ALI. CHE treatment significantly altered gut microbiota composition, particularly increasing levels of the beneficial bacterium *Barnesiella intestinihominis* (*p* < 0.05). In addition, CHE reversed metabolic disturbances and inhibited oxidative and inflammatory signaling pathways. These findings suggest that CHE is a natural hepatoprotective agent that prevents ALI by modulating gut microbiota, related metabolites, oxidative stress, and inflammation. This study provides new insights into CHE as a potential therapeutic approach for ALI.

## 1. Introduction

Drug-induced liver injury (DILI) denotes xenobiotic-mediated damage to the liver and biliary system, serving as the principal etiology of acute liver failure in Western countries [1,2,3]. Based on the presumed mechanisms of these compounds, drug-related hepatotoxicity is typically categorized into two types: intrinsic and idiosyncratic [4]. A primary example of intrinsic DILI is the hepatotoxicity of acetaminophen (APAP) [5]. During the COVID-19 pandemic, it gained immense popularity globally due to its notable antipyretic properties [6,7]. However, excessive dosing can lead to hepatotoxicity and even acute liver failure.

In recent years, research on the liver–gut axis has received considerable attention. Studies have shown that there is a close interaction between these two organs in normal physiology and disease [8]. Under normal circumstances, food and drugs are transported to the liver via the portal vein for metabolism or detoxification [9]. Conversely, bile acid (BA) and antimicrobial peptides produced in the liver are transported through the bile duct to the intestine, ultimately affecting the gut microbiota, intestinal epithelial cells, and immune cells [10]. Mounting evidence indicates that gut–liver axis dysfunction is associated with the occurrence and development of various liver diseases [11]. The gut microbiota plays a crucial role in the development of liver diseases [12,13]. Generally speaking, increased intestinal permeability and bacterial translocation may allow microbial metabolic products to reach the liver, thereby impeding BA metabolism and promoting intestinal motility disorders and systemic inflammation. All these conditions may induce an intestinal flora imbalance, which, in turn, further aggravates liver damage [14]. Notably, excess APAP leads to an imbalance of intestinal flora, which amplifies APAP-induced acute liver injury (ALI) [15]. Microbial metabolites are important mediators of host–microbial interactions. Some have beneficial effects on host physiology, such as short-chain fatty acids and secondary BA. In addition, tryptophan catalysts determine immune responses, for example, by binding to aromatic hydrocarbon receptors (AhRs). AhRs are abundantly present on the mucosal surface and, when activated, enhance the intestinal epithelial barrier function and regulate the immune response [16].

Chelerythrine (CHE), a benzophenanthridine alkaloid found in *Chelidonium majus* L., is a primary component of celandine. CHE has multiple effects, including antibacterial, anti-inflammatory, and antitumor activities [17,18,19,20,21,22,23,24]. Wei et al. [25] optimized the extraction process of CHE from *Chelidonium majus* L. and conducted in vitro antibacterial experiments on CHE. The results showed that CHE had certain inhibitory effects on the mycelium of various plant pathogenic fungi such as Muskmelon fusarium wilt. Li et al. [22] demonstrated in a mouse model that CHE exhibits anti-inflammatory effects by inhibiting TNF-α and NO production. Pěnčíková et al. [26] found that CHE inhibits LPS-induced inflammation produced by THP-1 cells. Danielewski et al. [27] reported that CHE inhibits the release of PGE2 in the Carrageenan Paw Edema Test in rats, inhibits TNF-α biosynthesis, and suppresses MMP-9 enzymatic activity. In a mouse pulmonary fibrosis model, CHE increased the expression of Nrf2, HO-1, and NQO1, and alleviated pulmonary fibrosis by activating the Nrf2/ARE pathway [28]. These results indicate that CHE plays an important role in antibacterial, anti-inflammatory, and antioxidant activities.

Liu and colleagues explored the activity of potential hepatoprotective components using virtual screening and machine learning, discovering that CHE and Buchanan A can enhance survival rates in cells damaged by APAP [29]. Current studies have indicated that Buchanan A exerts protective effects on the liver [30,31,32]; however, research on CHE’s impact on liver damage and its underlying mechanisms is still lacking. In a recent study, Ding and colleagues [33] discovered that CHE can protect against liver injury, which coincides remarkably with our research findings. However, the difference lies in that their study focused on the treatment of carbon tetrachloride-induced liver fibrosis in mice with CHE, whereas our focus is on the prevention of APAP-induced ALI with CHE. Importantly, we conducted systematic research and analysis from multiple perspectives, including gut microbiota and multi-omics approaches.

In our work, we demonstrated that CHE could alleviate APAP-induced ALI in mice. Furthermore, CHE was able to restore gut microbiota dysbiosis and increase the beneficial bacterium *Barnesiella intestinihominis* (*B. intestinihominis*), reducing the severity of ALI in mice. We combined metabolomics and transcriptomics to elucidate the potential mechanisms by which CHE exerts its therapeutic effects in APAP-induced ALI. The results of this study will lay the foundation for the development and utilization of antioxidant and anti-inflammatory drugs, while also contributing to new insights into the treatment and prevention of APAP-induced ALI.

## 2. Materials and Methods

### 2.1. Reagents and Chemicals

CHE was purchased from Chengdu DeSiTe Biological Technology Co., Ltd (Sichuan, China). APAP was purchased from Shanghai Aladdin Biochemical Technology Co., Ltd., (Shanghai, China) and N-acetylcysteine (NAC) was purchased from Solarbio Biotechnology Ltd (Beijing, China). The antibodies used in this study are listed in Supplementary Table S1. *B. intestinihominis* was purchased from the German Collection of Microorganisms (Braunschweig, German).

### 2.2. Animals and Experimental Design

Conventional wild-type C57BL/6J male mice (SPF, 20–22 g, 6–8 weeks old) were purchased from QIANHE BIOTECH Co., Ltd. (Permission code SCXK, 2019-0001, Jilin, China).

Experiment for the oral gavage of CHE. The mice were assigned randomly to five groups: (1) normal group; (2) APAP group; (3) APAP + NAC (15 mg/kg) group; (4) APAP + CHE (15 mg/kg) group; (5) APAP + CHE (30 mg/kg) group. The treatments were administered continuously for 7 days. Subsequently, the mice were given 350 mg/kg of APAP. After 24 h, serum, liver, and fecal samples were collected.

Experiment for *B. intestinihominis* intervention. Mice were treated with antibiotics via oral gavage for 7 days to deplete the gut microbiota [34]. The mice were assigned randomly to three groups: (1) APAP group; (2) APAP + *B. intestinihominis* (1 × 10^8^ CFU) group; (3) APAP + *B. intestinihominis* (1 × 10^8^ CFU) + CHE (30 mg/kg) group. *B. intestinihominis* and CHE were administered to the mice via gavage for 7 days. Subsequently, the mice received 350 mg/kg of APAP. After 24 h, serum and liver samples were collected.

### 2.3. Biochemical Assays

All biochemical tests were conducted in accordance with the instructions, and the OD values were measured using a microplate reader. ALT, AST, IL-6, IL-1β, TNF-α, MPO, MDA, H_2_O_2_, CAT, SOD, and GSH kits were purchased from Nanjing Jiancheng Bioengineering Institute (Nanjing, China) or Beyotime Biotechnology (Shanghai, China).

### 2.4. Quantitative Real-Time PCR

Total RNA was extracted from liver samples using TRIzol reagent (California, CA, USA) in accordance with the manufacturer’s instructions, and its integrity was evaluated before subsequent experiments. cDNA was synthesized using the HiScript II reverse Transcription Kit (Nanjing, China) with an equal amount of RNA as the template. Real-time fluorescence quantitative PCR was performed on the ABI QuantStudio 3 platform (Waltham, MA, USA) using SYBR Green dye and following the reported best practice [35]. To ensure measurement reliability, three technical replicates were set for each biological sample [36]. Normalizing with GAPDH as the internal reference, the relative expression level was calculated using the 2^−ΔCT^ method. The primer sequence is shown in Supplementary Table S2.

### 2.5. Histology

First, liver tissue was placed in a 10% formaldehyde buffer solution and was then dehydrated and embedded in paraffin for sectioning. Finally, the sections were deparaffinized with xylene, rehydrated, and stained.

### 2.6. 16S rRNA Gene Expression Analysis

DNA pyrosequencing targeting the V3–V4 hypervariable region of the 16S rRNA gene was performed using the Illumina platform. The sequencing results, obtained through operational taxonomic unit clustering, species annotation, and abundance analysis, provided insights into the species composition of the samples.

### 2.7. Metabolomics Analysis

Liver tissue (about 80 mg) was dispersed in 200 μL of water and mechanically processed for 60 s. Next, 800 μL of a 1:1 (*v*/*v*) methanol–acetonitrile solvent was introduced, and the extract underwent two rounds of chilled sonication, each for 30 min. Proteins were removed by precipitation at −20 °C for 1 h, followed by centrifugation at 4 °C for 20 min. The clarified supernatant was collected and freeze-dried, and the residue was reconstituted to generate metabolite extracts, which were kept at −80 °C until measurement. Untargeted metabolite profiling was conducted by ultra-high-performance liquid chromatography coupled with quadrupole time-of-flight mass spectrometry (UHPLC-Q-TOF/MS). Separation was achieved on an Agilent 1290 Infinity LC platform using a hydrophobic interaction column, operated at 25 °C with a 0.30 mL/min flow. Eluent A consisted of water with 25 mmol/L ammonium acetate and 25 mmol/L ammonia, while eluent B was acetonitrile. Detection employed electrospray ionization in both positive and negative polarities. Feature identification relied on high-accuracy mass tolerance (<25 ppm) together with MS/MS spectral agreement. Before modeling, data were processed via Pareto scaling. Orthogonal partial least squares–discriminant analysis (OPLS-DA) was performed in R (v3.3.1) to improve class discrimination by removing variation unrelated to grouping.

### 2.8. RNA Sequencing

Total RNA was extracted from liver tissue using Trizol reagent. RNA purification, reverse transcription, library construction, and sequencing were performed by Shanghai Meiji Biotechnology Co., Ltd., (Shanghai, China) according to the manufacturer’s instructions (Illumina, San Diego, CA, USA).

### 2.9. Western Blot

Liver tissue was homogenized in RIPA lysis buffer. The protein concentration was determined using a BCA protein assay kit, the mixture was boiled in a DualColor sample buffer for proteins at 98 °C, equal amounts of protein were separated using 12% SDS-PAGE, and the amounts were then separated to a PVDF membrane (200 mA, 80 min). The sample was incubated with primary and secondary antibodies at 4 °C overnight (dilution 1:1000). Then, the protein bands were detected using enhanced chemiluminescence (ECL). Finally, a quantitative analysis was performed using ImageJ-1.51j8.

### 2.10. Statistical Analysis

In this study, we used Student’s t-test, Kruskal–Wallis test, and ANOVA. All graphs were generated using GraphPad Prism 7. Statistical significance was indicated according to the following criteria: (^#^ or *) *p* < 0.05, (^##^ or **) *p* < 0.01, and (^###^ or ***) *p* < 0.001.

## 3. Results

### 3.1. Preventive Effects of CHE on ALI in Mice

**Figure 1:**
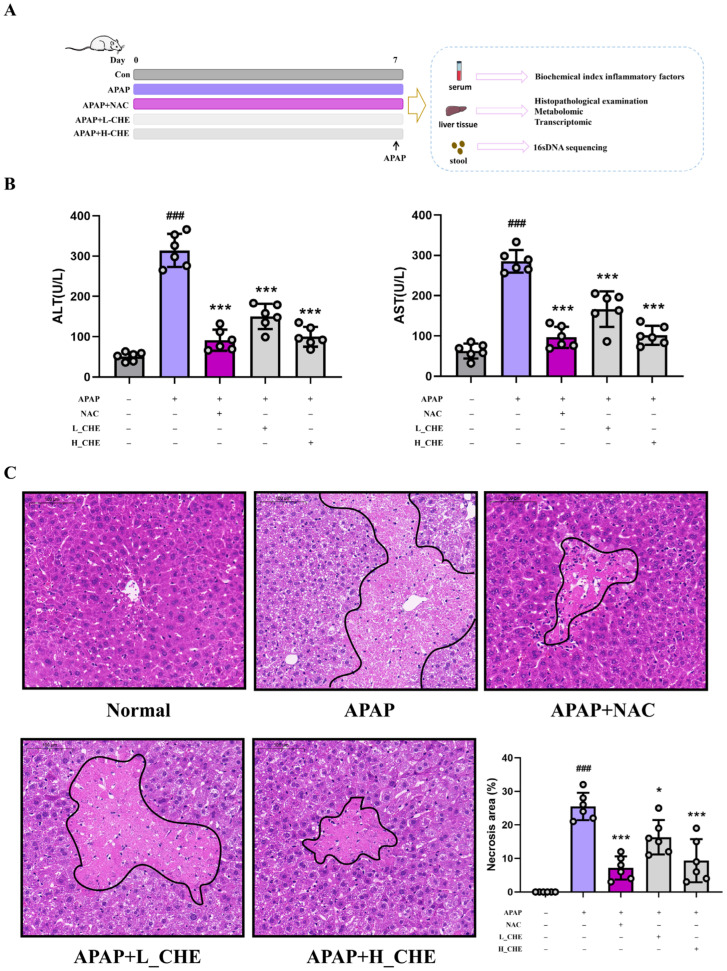
Effect of CHE on ALI induced by APAP. (**A**) Flowchart of mouse experiments. (**B**) Serum ALT and AST activities. (**C**) H&E-stained liver sections at 400× magnification. Data are expressed as mean ± SD (*n* = 6 each group). ^#^ Compared to the normal group. * Compared to the APAP group. (*) *p* < 0.05; (^###^ or ***) *p* < 0.001.

As shown in the experimental flowchart in Figure 1A, an ALI model induced via APAP was established 7 days after administering the CHE treatment. Figure 1B demonstrated that CHE significantly reduced the APAP-induced elevations in ALT and AST. Liver injury resulted in marked vacuolization of hepatocytes with massive inflammatory cell infiltration. After CHE treatment, both the inflammatory infiltration and other damage were notably alleviated (Figure 1C). These results lay the groundwork for further research into the mechanisms by which CHE modulates ALI.

### 3.2. CHE Alleviates APAP-Induced Inflammation and Oxidative Stress Injury

**Figure 2:**
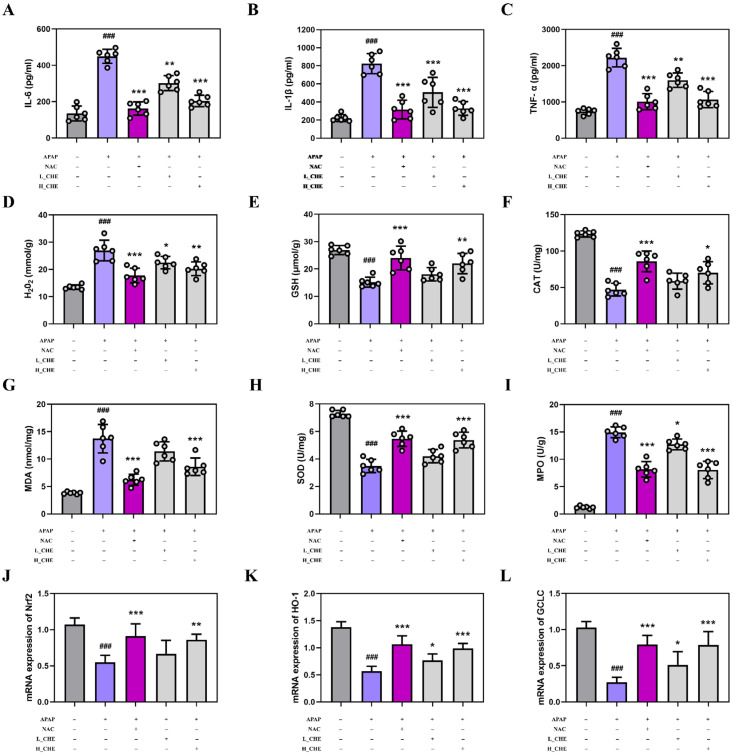
Effects of CHE on APAP-induced inflammation and oxidative stress. (**A**–**C**) Serum inflammatory factor IL-6, IL-1β, and TNF-α levels. (**D**–**I**) Hepatic H_2_O_2_, GSH, CAT, MDA, SOD, and MPO contents. (**J**–**L**) Hepatic Nrf2, HO-1, and GCLC mRNA expression. Data are expressed as mean ± SD (*n* = 6 each group). ^#^ Compared to the normal group. * Compared to the APAP group. (*) *p* < 0.05, (**) *p* < 0.01, and (^###^ or ***) *p* < 0.001.

It is well known that excessive APAP can induce inflammation and oxidative stress. Compared to the normal group, the APAP group showed significantly elevated levels of IL-1β, IL-6, and TNF-α in mouse serum, as well as significant changes in hepatic H_2_O_2_, GSH, CAT, MDA, SOD, MPO, Nrf2, HO-1, and GCLC. However, the CHE group reversed these APAP-induced inflammatory and oxidative stress changes (Figure 2A–L). These results clearly demonstrate that CHE reduced APAP-induced inflammatory and oxidative stress damage.

### 3.3. CHE Restores Gut Microbiota Dysbiosis in ALI Mice

**Figure 3:**
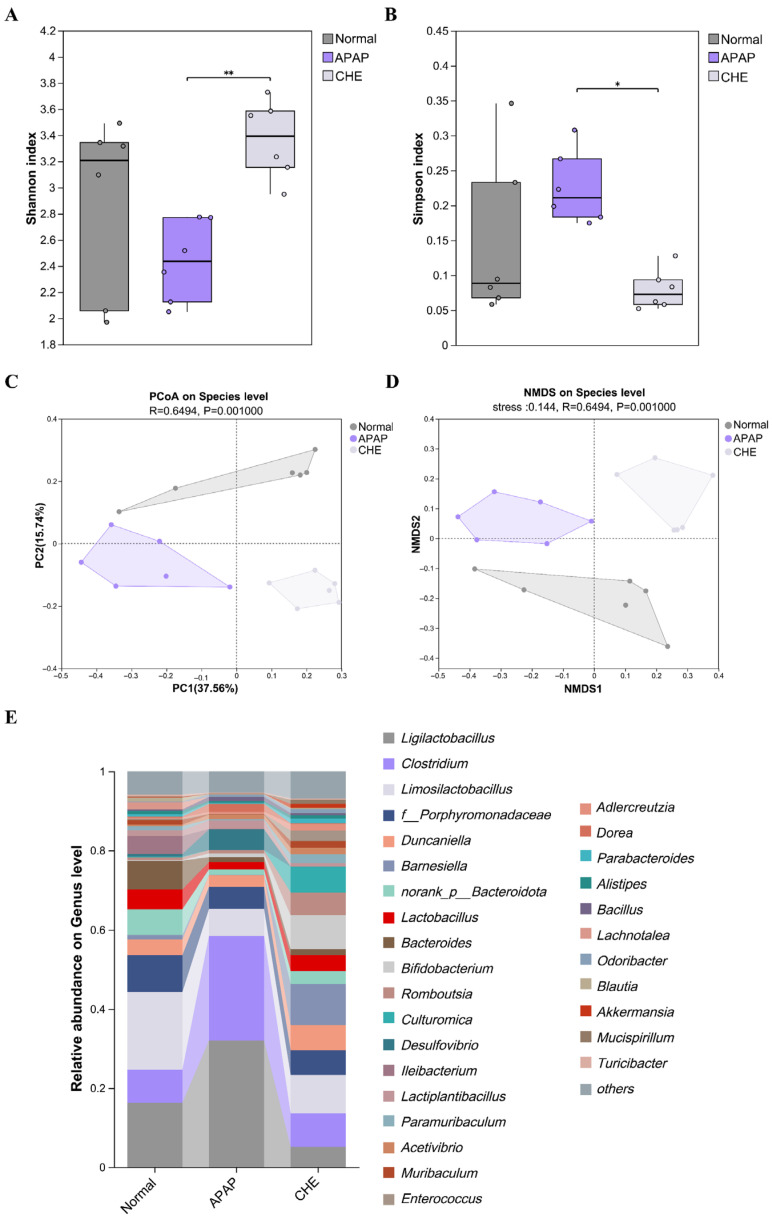
The impact of CHE on the recovery of intestinal microbiota dysbiosis in ALI mice. (**A**,**B**) The Shannon and Simpson indices. (**C**,**D**) PCoA and NMDS analysis. (**E**) An overview of the genus level of microorganisms. Data are expressed as mean ± SD (*n* = 6 each group). (*) *p* < 0.05; (**) *p* < 0.01.

Considering the crucial role of gut microbiota in ALI [37], we researched the impact of CHE on gut microbiota composition. The use of Shannon and Simpson indices for alpha diversity analysis revealed changes in the diversity and richness of the CHE community (Figure 3A,B). Using PCoA and NMDS for beta diversity analysis, significant separation among groups was observed, confirming CHE’s regulatory effect on gut microbiota diversity (Figure 3C,D). At the genus level, the model group exhibited an increase in the abundance of *Clostridium* and a decrease in the abundance of *Limosilactobacillus*, while CHE reversed these changes in abundance (Figure 3E). In summary, our findings demonstrate that CHE recovers microbial dysbiosis and enhances the presence of beneficial bacteria.

### 3.4. B. intestinihominis Is the Key Functional Strain Through Which CHE Regulates the Gut Microbiota

**Figure 4:**
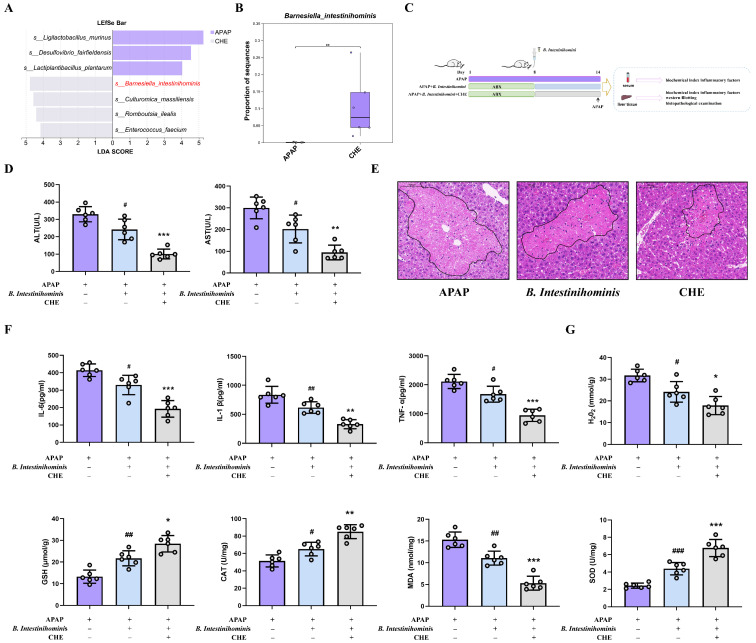
The preventive effect of *B. intestinihominis* on ALI. (**A**) LEfSe analysis. (**B**) Relative abundance of *B. intestinihominis*. (**C**) Experimental design for Oral *B. intestinihominis*. (**D**) Serum ALT and AST activities. (**E**) Liver H&E staining (100 μm). (**F**) Levels of serum inflammatory factors IL-6, IL-1β, and TNF-α. (**G**) Hepatic GSH, CAT, H_2_O_2_, MDA, and SOD contents. Data are expressed as mean ± SD (*n* = 6 each group). (^#^ or *) *p* < 0.05; (^##^ or **) *p* < 0.01; and (^###^ or ***) *p* < 0.001.

To identify bacterial species contributing to the mitigation of ALI by CHE, we analyzed the effects of CHE treatment on the gut microbiota at the species level. Notably, *B. intestinihominis* was enriched, and LEfSe identified it as a marker species in the CHE-treated group (Figure 4A). The abundance of *B. intestinihominis* in the CHE group is significantly up-regulated (Figure 4B). *B. intestinihominis* has been identified as a beneficial bacterium associated with liver injury [38]. Based on this, we hypothesize that *B. intestinihominis* plays a pivotal role in mediating CHE’s hepatoprotective effects. Interestingly, our results indicate that CHE significantly increased the activity of *B. intestinihominis* in vitro (Figure S1). In vivo experiments further confirmed that *B. intestinihominis* can alleviate APAP-induced inflammation and injury, reducing the levels of ALT, AST, IL-6, IL-1β, TNF-α, H_2_O_2_, and MDA, while increasing the levels of GSH, CAT, and SOD (Figure 4D–G). Collectively, these findings suggest that *B. intestinihominis* is a key functional strain in the gut microbiota modified by CHE, contributing to its protective effects against ALI.

### 3.5. Effects of CHE on Liver Metabolic Levels

**Figure 5:**
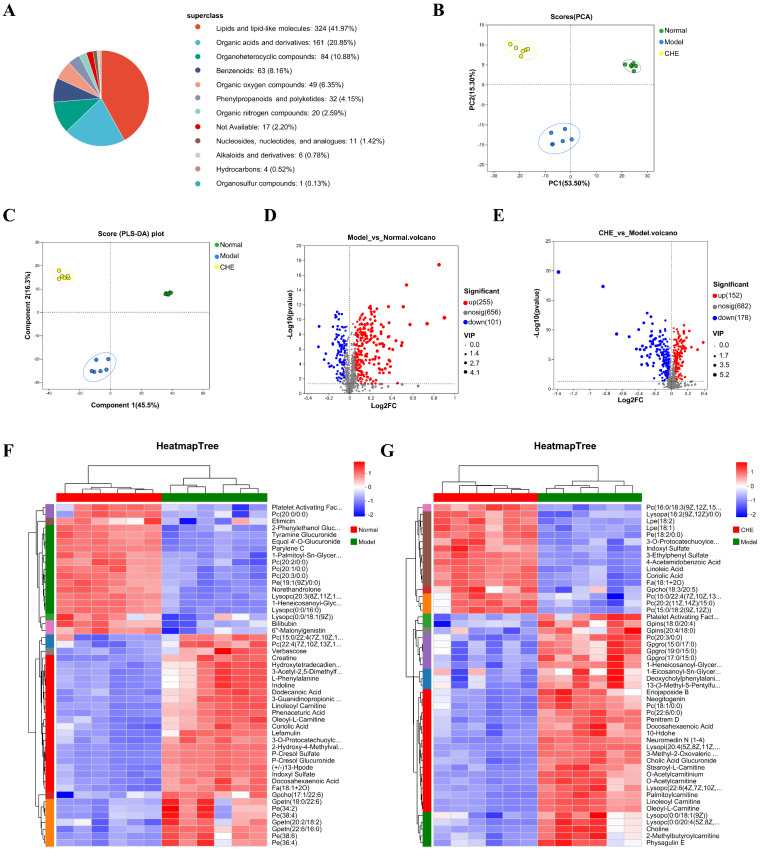
The influence of CHE on the non-targeted metabolome of serum. (**A**) Metabolite classification based on the HMDB database. (**B**) PCA analysis of metabolites. (**C**) OPLS-DA analysis of metabolites. (**D**) Volcano map analysis of differential metabolites between positive and negative ions in APAP vs. normal. (**E**) Volcano map analysis of differential metabolites of positive and negative ions in APAP vs. CHE. (**F**,**G**) Cluster heatmap of DAMs.

The HMDB comprises a comprehensive collection of 772 annotated metabolites, providing valuable insights into human biochemistry. The majority of these metabolites are categorized into several key classes, with lipids and lipid-like molecules representing the largest group at 41.97%. Following this, organic acids and derivatives account for 20.85%, organoheterocyclic compounds make up 10.88%, and benzenoid compounds constitute 8.16%. This classification, illustrated in Figure 5A, highlights the diverse range of metabolites present in the human body and underscores the importance of these compounds in various metabolic processes. PCA and PLS-DA analyses indicated clear separation between the groups (Figure 5B,C). These findings underscore the potential of using metabolomics to elucidate the biochemical alterations associated with different experimental conditions. The volcano plots depicting the differential regulation of metabolites between the APAP group and the normal group are illustrated in Figure 5D. A total of 255 metabolites were found to be up-regulated and 101 metabolites down-regulated in the APAP group compared to the normal group. Additionally, Figure 5E presents the volcano plots comparing the APAP group to the CHE group, revealing that the CHE group exhibited an up-regulation of 152 metabolites and a down-regulation of 178 metabolites. Furthermore, the clustering heat maps for the comparisons of APAP versus normal and APAP versus CHE groups are shown in Figure 5F and Figure 5G, respectively. The clustering results indicate a clear distinction between groups, with significant differences observed in metabolite levels, underscoring the impact of APAP administration and its modulation by CHE treatment.

### 3.6. CHE’s Regulatory Effect on ALI Metabolism

**Figure 6:**
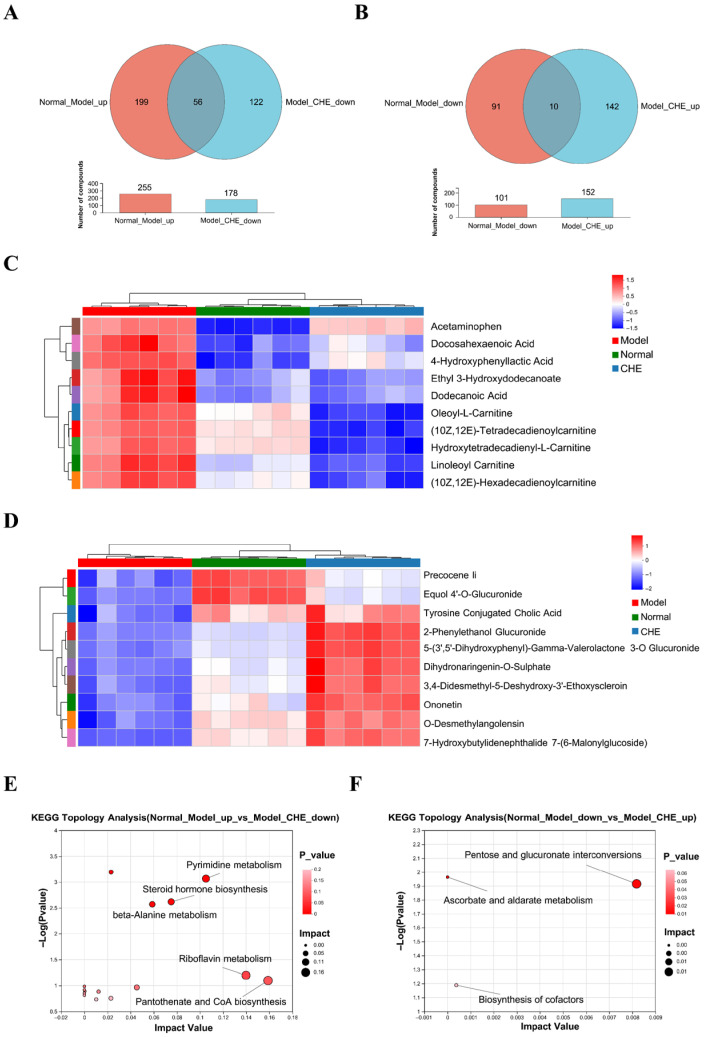
The regulatory effect of CHE on DAMs. (**A**,**B**) Venn diagram of DAMs. (**C**,**D**) Cluster heatmap of DAMs. (**E**,**F**) KEGG enrichment analysis of DEGs.

To further investigate the mechanism of CHE protection against ALI, we determined the differential abundance metabolites (DAMs) before and after CHE treatment. Figure 6A,B illustrate the identification of 56 DAMs up-regulated by APAP but down-regulated by CHE, along with 10 DAMs down-regulated by APAP and subsequently up-regulated by CHE. Cluster analysis of DAMs across experimental groups was performed (Figure 6C,D). Following this, enrichment analysis of the identified DAMs was conducted to determine the significantly enriched metabolic pathways. The DAMs promoted by APAP but inhibited by CHE were primarily enriched in pathways related to “Pyrimidine metabolism,” “Steroid hormone biosynthesis,” “beta-Alanine metabolism,” “Riboflavin metabolism,” and “Pantothenate and CoA biosynthesis” (Figure 6E). Additionally, the DAMs suppressed by APAP but up-regulated by CHE displayed significant enrichment in pathways associated with “Pentose and glucurorate interconversions”, “Ascorbate and aldarate metabolism”, and “Biosynthesis of cofactors” (Figure 6F).

### 3.7. Effects of CHE on Liver Gene Expression Levels

**Figure 7:**
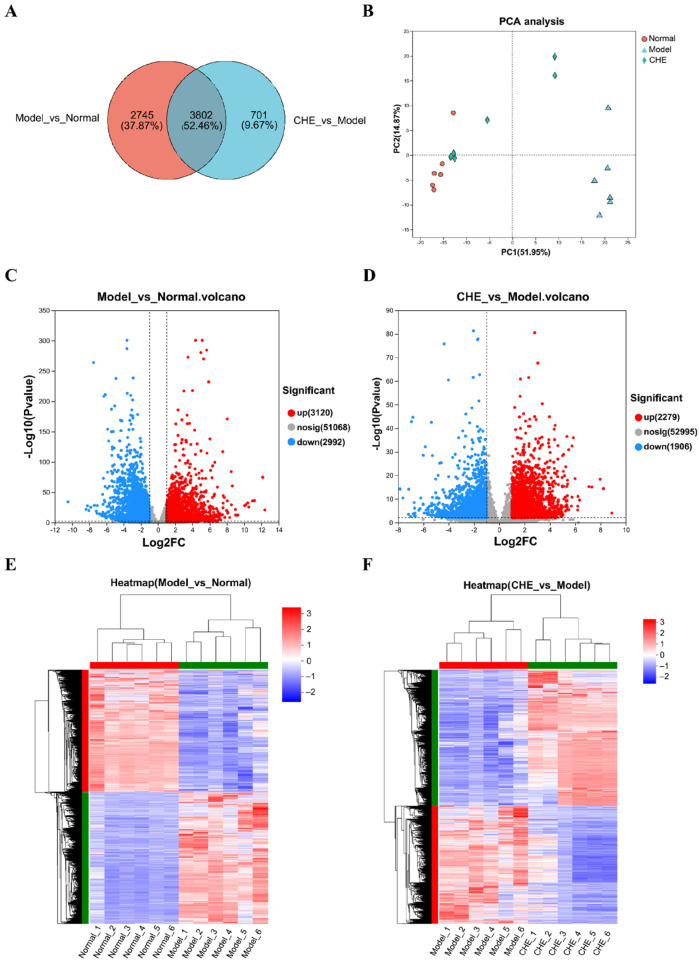
Effects of CHE on hepatic transcriptome. (**A**) Venn diagram of DEGs. (**B**) PCA score plot of transcriptomics analysis. (**C**,**D**) Volcano plot of significant DEGs. (**E**,**F**) Clustering heatmap of DEGs.

Transcriptome sequencing to study the mechanism of CHE in preventing ALI. In this study, we analyzed 18 samples through RNA sequencing to investigate the effects of CHE on ALI induced by APAP. Our comparative analysis of the two transcriptomes uncovered considerable overlap between the identified genes, with 3802 genes common to both comparisons (Figure 7A). The PCA score plot demonstrated distinct differences in liver transcriptomes among the experimental groups (Figure 7B). Our findings, illustrated in Figure 7C, revealed the identification of 6112 genes when comparing the APAP group to the normal group, comprising 3120 up-regulated genes and 2992 down-regulated genes. Furthermore, as shown in Figure 7D, a comparison between the CHE group and the APAP group identified 4185 genes, which included 2279 up-regulated genes and 1906 down-regulated genes. Clustering analyses in Figure 7E,F demonstrated that all three groups clustered tightly, indicating homogeneity within each group, while pronounced differences in gene expression were evident between the groups. Importantly, these results highlight significant inter-group variances in gene expression, with minimal intra-group discrepancies. Notably, pretreatment with CHE resulted in the modulation of certain genes in the APAP group, effectively aligning their expression profiles with those of the normal group. This suggests that CHE exerts a protective effect against alterations in gene expression during APAP-induced ALI. These results underscore the potential of CHE to reverse the gene expression changes instigated by the APAP administration. Collectively, this study elucidates the intricate relationship between transcriptomic variations and the protective mechanisms conferred by CHE, highlighting its therapeutic potential in mitigating liver injury.

### 3.8. Key Regulatory Role of CHE in Transcriptional Levels

**Figure 8:**
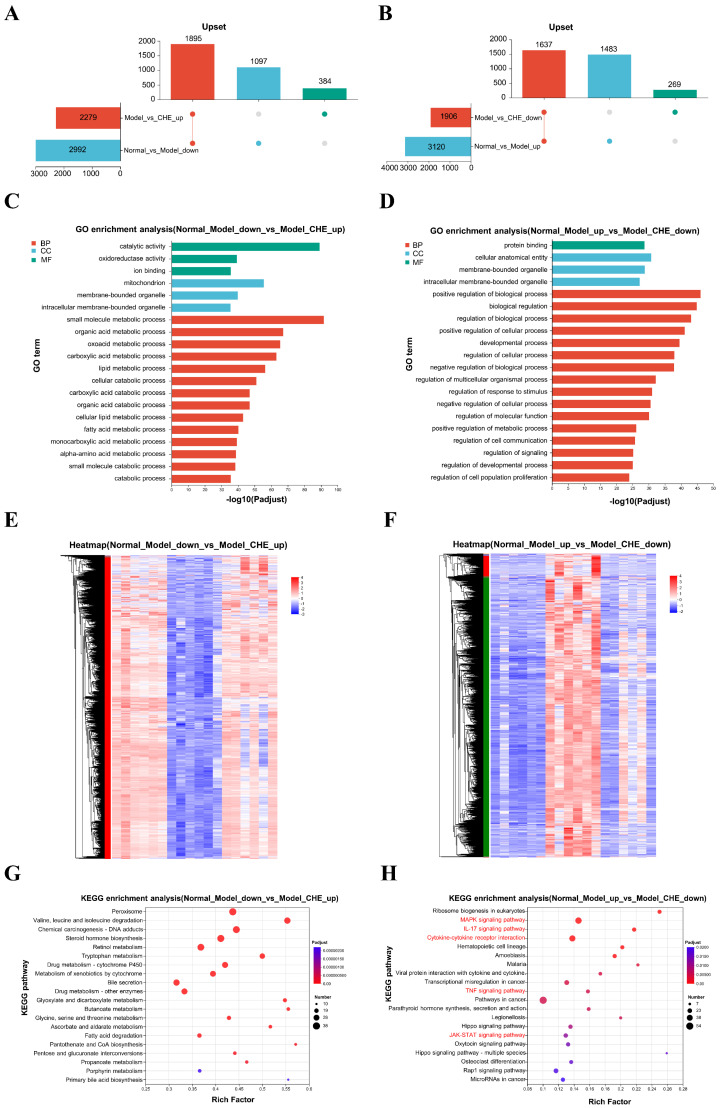
The regulatory effect of CHE on DEGs. (**A**,**B**) Upset plot of DEGs. (**C**,**D**) GO functional annotation analysis of DEGs. (**E**,**F**) Clustering heatmap of DEGs. (**G**,**H**) KEGG enrichment analysis of DEGs.

To elucidate the critical pathways through which CHE influences APAP-induced ALI, we generated two genomic datasets. As illustrated in Figure 8A, APAP suppresses the expression of 1895 genes, while CHE enhances it. Conversely, our analysis revealed that 1637 genes are elevated following APAP treatment but diminished with CHE intervention (Figure 8B). The differentially expressed genes (DEGs) down-regulated by APAP and up-regulated by CHE are significantly associated with “catalytic activity,” “mitochondrion,” “small molecule metabolic processes,” and “organic acid metabolic processes” (Figure 8C). Similarly, the DEGs up-regulated by APAP and down-regulated by CHE predominantly relate to categories such as “biological regulation,” “regulation of biological processes,” “cellular anatomical entity,” and “protein binding” (Figure 8D). Figure 8E,F showed distinct clustering of normal, APAP, and CHE samples. KEGG enrichment analysis indicates that the DEGs down-regulated by APAP but up-regulated by CHE show significant enrichment in pathways related to “Valine, leucine and isoleucine degradation,” “Retinol metabolism,” “Drug metabolism-other enzymes,” and “Drug metabolism-cytochrome P450,” indicating that CHE’s hepatoprotective effect may be mediated through drug metabolism (Figure 8G). Conversely, the DEGs up-regulated by APAP and down-regulated by CHE are notably enriched in various inflammation and apoptosis-related pathways, including “Cytokine-cytokine receptor interaction,” “MAPK signaling pathway,” “Pathways in cancer,” “TNF signaling pathway,” “JAK-STAT signaling pathway,” and “IL-17 signaling pathway” (Figure 8H). These results indicate that CHE regulates the transcriptional changes induced by APAP.

### 3.9. Integrated Analysis of CHE Protection Against APAP-Induced ALI

**Figure 9:**
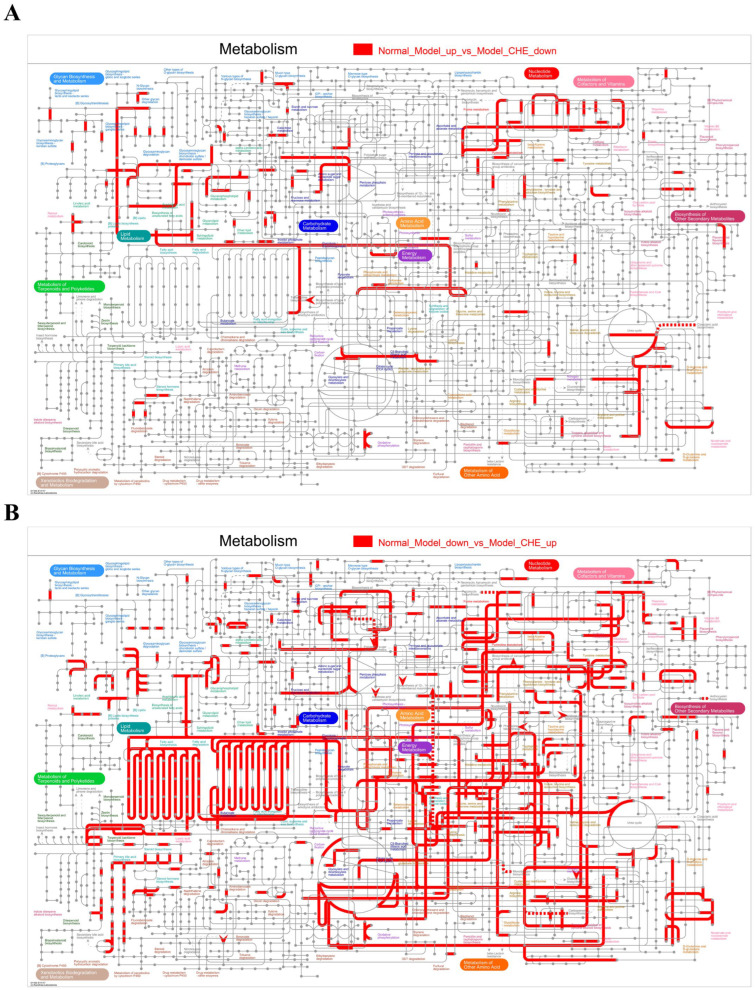
Integrated metabolome and transcriptome analysis. (**A**,**B**) Distribution of CHE-reversed APAP-induced DEGs and DAMs in KEGG metabolic pathway maps.

We further integrated the DEG and DAM for analysis. DEGs and DAMs that were up-regulated by APAP and down-regulated by CHE were significantly enriched in metabolic pathways such as “Nucleotide Metabolism,” “Carbohydrate Metabolism,” “Amino Acid Metabolism,” and “Energy Metabolism” (Figure 9A). Conversely, they showed significant enrichment in pathways related to “Nucleotide Metabolism,” “Amino Acid Metabolism,” and “Lipid Metabolism” (Figure 9B). These findings indicate that CHE administration effectively alleviates the metabolic abnormalities induced by APAP in liver tissues.

### 3.10. CHE Inhibits Oxidative Stress and Pro-Inflammatory Signaling in ALI

**Figure 10:**
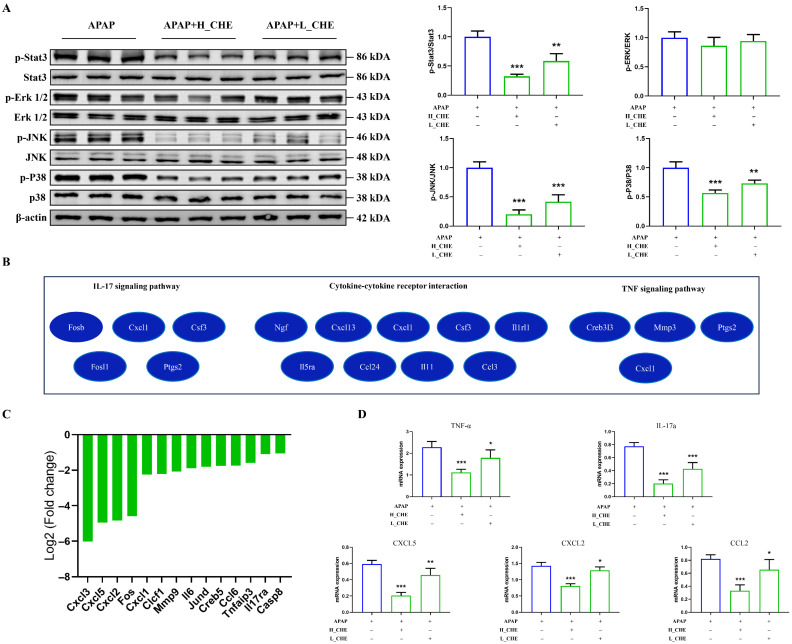
CHE inhibits the liver oxidative stress and pro-inflammatory pathways. (**A**) Western blot analysis of p-STAT3, p-ERK, p-JNK, p-p38, STAT3, ERK, JNK, p38, and β-actin protein expression. (**B**) DEGs of IL-17 signaling pathway, cytokine–cytokine receptor interaction pathway, and TNF signaling pathway were revealed in the enrichment analysis network. (**C**) In the APAP model, mouse inflammatory response and autoimmune PCR arrays showed significantly down-regulated expression of 14 transcripts between the normal and CHE groups. (**D**) Quantitative RT-PCR was used to verify the expression changes in Ccl2, Cxcl2, Cxcl5, TNF-a, and IL-17a. * *p* < 0.05, ** *p* < 0.01, and *** *p* < 0.001.

To gain deeper insights into the molecular mechanisms underlying the protective effects of CHE in liver injury, we conducted RNA sequencing of liver tissue. Our KEGG enrichment analysis identified the MAPK and JAK signaling pathways as significantly down-regulated by CHE treatment. Correspondingly, we observed that CHE treatment suppressed the expression of p-STAT3, p-JNK, and p-p38 in the liver tissue (Figure 10A). Thus, CHE inhibits the MAPK and JAK signaling pathways associated with liver injury. Indeed, our RNA sequencing results indicate that CHE can attenuate pro-inflammatory IL-17 signaling, TNF signaling, and cytokine–cytokine receptor interactions (Figure 10B). Consistently, our analysis of the inflammatory response and autoimmune PCR array in the APAP model revealed a reduction in 14 genes encoding cytokines and their receptors following CHE treatment (Figure 10C). Quantitative PCR validated these results, demonstrating down-regulation of mRNA levels for Ccl2, Cxcl2, Cxcl5, TNF-α, and IL-17a in liver tissue post-CHE treatment (Figure 10D). Overall, the effects of CHE on liver injury are mediated through oxidative stress and pro-inflammatory factor-signaling cascades.

## 4. Discussion

Currently, there are limited treatments for APAP-induced ALI, and liver transplantation may be considered in advanced stages of liver injury. Therefore, preventive approaches are important to reduce the risk of APAP. Natural products have the characteristic of multi-targeting and can exert therapeutic effects through various mechanisms. They are widely available and resource-rich, giving them a unique advantage in the development of liver-protective medications. In this study, we confirmed the hepatoprotective effect of CHE on APAP model mice. In this model, CHE significantly reduced oxidative stress and inflammation and improved APAP-induced ALI. Importantly, doses of CHE that exert liver protection are safe and well tolerated (Figure S2).

Excessive accumulation of APAP toxicity leads to liver damage, with ALI including oxidative stress and inflammation [39]. We found that excess APAP resulted in marked dilatation of the hepatic lobules and central portal vein and marked vacuolization of hepatocytes with marked inflammatory cell infiltration in mice. After pretreatment with CHE, the inflammatory infiltration and other damages were significantly reduced. The abnormal alterations of oxidative stress and inflammatory factors such as ALT, AST, IL-6, IL-1β, TNF-α, MPO, MDA, H_2_O_2_, CAT, SOD, and GSH were also reversed. This suggests that CHE prevents ALI through its antioxidant properties and by inhibiting inflammation.

Considering the importance of gut microbiota in the pathogenesis of ALI, we analyzed different mouse fecal samples via 16S rDNA sequencing. We found that CHE pretreatment restored gut flora disruption, increased flora diversity, and reversed APAP-induced reduction in beneficial bacteria. In a recent study, *B. intestinihominis* bacteria were found to ameliorate hypertension and hepatic metabolic disorders, and *B. intestinihominis*-derived acetate enhanced fibroblast growth factor 21 by increasing H3 K27 acetylation of the fibroblast growth factor 21 promoter through inhibition of histone deacetylase 9. Importantly, we found that oral administration of *B. intestinihominis* ameliorated APAP-induced hepatic injury and inflammatory bursts and significantly inhibited oxidative stress injury. Therefore, we hypothesized that *B. intestinihominis* is a key functional strain of the CHE-protective gut microbiota against ALI.

Given that the development of ALI leads to metabolic disturbances, we analyzed the interaction between CHE and metabolites. Metabolomic results indicate that CHE can inhibit metabolic disorders. Our results suggest that APAP inhibits “Pentose and glucurorate interconversions”, “Ascorbate and aldarate metabolism”, and “Biosynthesis of cofactors” metabolic pathways in the liver, while CHE pretreatment enhanced the ability of the mouse liver to regulate these metabolic pathways. Among the many differential metabolites, 2-Phenylethanol Glucuronide is the most enriched. Research shows that approximately 80–90% of APAP is metabolized through glucuronidation and sulfation, catalyzed by glucuronosyltransferases and sulfotransferases, resulting in non-toxic metabolites that are subsequently transferred to the plasma and excreted in urine [40]. CHE can restore elevated levels of 2-Phenylethanol Glucuronide, indicating that the liver is actively glucuronidating 2-phenylethanol, typically to promote its excretion. This reflects enhanced hepatic metabolic function, and higher levels of 2-Phenylethanol Glucuronide may indicate that the liver is attempting to protect itself by increasing detoxification processes.

To further explore CHE’s mechanism of action in the treatment of ALI, we conducted transcriptome sequencing analysis of the liver tissues from each group of mice. Intact-tissue RNA-seq shows that CHE inhibits oxidative stress and pro-inflammatory pathways in liver injury. It mainly includes the MAPK and JAK pathways. The MAPK and JAK signaling pathways play important roles in liver injury, and the MAPK pathway is highly enriched. CHE reduces the expression of oxidative stress and pro-inflammatory IL-17a, Ccl2, Cxcl2, Cxcl5, and TNF-α cytokines that drive immune cell chemotaxis. Research has demonstrated that both IL-17 and TNF signaling pathways can activate downstream MAPK signaling pathways (particularly JNK and p38), enhancing inflammatory gene transcription and post-transcriptional stability. IL-17 and TNF signaling pathways exhibit synergistic effects and can jointly activate MAPK signaling pathways, resulting in additive inflammatory output. While not primarily operating through the JAK-STAT signaling pathway, they can indirectly trigger JAK-STAT signaling by inducing secondary cytokines (such as IL-6), thereby further amplifying the inflammatory circuit [41,42,43,44,45,46]. These findings suggest that CHE treatment inhibits oxidative stress and pro-inflammatory signaling to prevent ALI. Meanwhile, our research demonstrates that CHE pretreatment restored the regulation of pathways related to “Drug metabolism-other enzymes” and “Drug metabolism-cytochrome P450” in APAP-induced ALI, suggesting that CHE may alleviate APAP-induced liver injury through its effects on drug metabolism. Our study provides insight into how CHE protects the liver from oxidative stress and inflammation in multiple ways and informs future strategies for utilizing CHE to prevent liver injury.

With excessive APAP use, increased NAPQI leads to hepatic GSH depletion and formation of protein adducts, thereby exacerbating oxidative stress and cellular injury [47]. As a GSH precursor, NAC can restore hepatic GSH and exert protective effects by scavenging NAPQI and preventing protein adduct formation [48]; importantly, this study shows that both NAC and CHE significantly increased GSH levels and corrected APAP-induced abnormalities in H_2_O_2_, CAT, MDA, SOD, MPO, Nrf2, HO-1, and GCLC (Figure 2D–L), with comparable efficacy between the two. These results clearly indicate that CHE, like NAC, exerts antioxidant protection against ALI. In addition, NAC has mechanisms that modulate inflammatory processes, which is reflected in our experimental finding that CHE and NAC have equivalent anti-inflammatory effects (Figure 2A–C). NAC is the only drug approved by the FDA for the treatment of APAP-induced ALI [49]. However, NAC has many drawbacks, including limited efficacy, low oral bioavailability of supplemental formulations, and adverse reactions [50,51,52]. Some herbal medicines are even more effective than NAC in treating ALI [53]. We found that CHE can up-regulate GSH, thereby enhancing APAP detoxification, which suggests that combining GSH-increasing herbs with NAC could improve the clinical efficacy of ALI treatment.

Currently, hepatoprotective drugs such as the synthetic strong pro-oxidant scavenger tiopronin may cause cancer and inflammation with long-term use; glycyrrhizic acid can induce edema, hypertension, and hypokalemia; S-adenosyl-L-methionine may cause gastrointestinal discomfort, insomnia, and agitation; and silymarin can produce gastrointestinal upset, diarrhea, and rashes. Compared to traditional hepatoprotective drugs, CHE, as a natural chemical compound, demonstrates moderate efficacy in regulation, offers multiple therapeutic effects, and has no side effects. Although we observed that CHE can protect against ALI and explored related mechanisms, the effects of *B. intestinihominis* metabolites on ALI and the clinical application of CHE together with *B. intestinihominis* in ALI patients still require further in-depth study and validation in the future.

## 5. Conclusions

In summary, our study demonstrates for the first time the in vivo preventive effect of CHE on ALI. CHE can restore gut microbiota imbalance, increase the levels of beneficial bacteria, regulate metabolic disturbances, protect against ALI, and inhibit pro-inflammatory pathways. Our study provides an in-depth exploration of the complex interactions among CHE-regulated gut microbiota, metabolism, oxidative stress, and inflammatory pathways, providing guidance for future use of CHE in protecting against ALI.

## Data Availability

The original contribution presented in this study is included in the article/Supplementary Material, and the raw 16S rRNA gene and RNA-seq data are freely available in the NCBI database under accession no. PRJNA1288401 and PRJNA1284048. Data will be available on reasonable request.

## Supplementary Materials

The following supporting information can be downloaded at https://www.mdpi.com/article/10.3390/antiox14091063/s1. Figure S1: *B. intestinihominis* was treated with CHE or blank control for 48 hours, and bacterial growth was then analyzed by measuring turbidity at 600 nm; Figure S2: Pathological effects of high-dose CHE treatment on the lung, liver, kidney, and spleen of mice. Table S1: Antibodies used in this study; Table S2: Primer sequence.

## Abbreviations

The following abbreviations are used in this manuscript:

ALI	Acute liver injury
APAP	Acetaminophen
*B. intestinihominis*	*Barnesiella intestinihominis*
DAMs	Differential abundance metabolites
DEGs	Differentially expressed genes
DILI	Drug-induced liver injury
GAPDH	Glyceraldehyde 3-phosphate dehydrogenase
H&E	Hematoxylin and eosin
HMDB	Human Metabolome Database
KEGG	Kyoto Encyclopedia of Genes and Genomes
CHE	Chelerythrine
LEfSe	Linear discriminant analysis effect size
NAC	N-acetylcysteine
PCA	Principal component analysis
PCR	Polymerase chain reaction
