# Supplementary material for: Chelerythrine Protects Against Acetaminophen-Induced Acute Liver Injury: Insights from Gut Microbiota and Multi-Omics Analysis
Source: Antioxidants (Basel). 2025 Aug 29;14(9):1063. doi: 10.3390/antiox14091063 (PMC12466735; doi:10.3390/antiox14091063)
Supplement: Supplementary file 1 [file antioxidants-14-01063-s001.zip › antioxidants-3780221-supplementary.pdf]

Supporting Information

Chelerythrine Protects Against Acetaminophen-Induced Acute Liver Injury: Insights from Gut Microbiota and Multi-Omics Analysis

Jinlong Liu †, Yanfei Zhang †, Hao Wu, Pan Yang, Wenlong Wang, Chenliang Li, Hong Cao, Jinying Wu and Xin Sun *

School of Pharmaceutical Sciences, Jilin Medical University, No.5, Jilin Street, Fengman District, Jilin 132013, China; jinlongliu@jlmu.edu.cn (J.L.); zyf20181812@163.com (Y.Z.); haowu@jlmu.edu.cn (H.W.); 15699571396@163.com (P.Y.); 13944674691@163.com (W.W.); lcl07292025@163.com (C.L.); 13234351232@163.com (H.C.); 13944628227@163.com (J.W.)

* Correspondence: sunxin@jlmu.edu.cn

† These authors contributed equally to this work.

This file includes:

Figs. S1 to S2

Tables. S1 to S2

Supplementary Figures

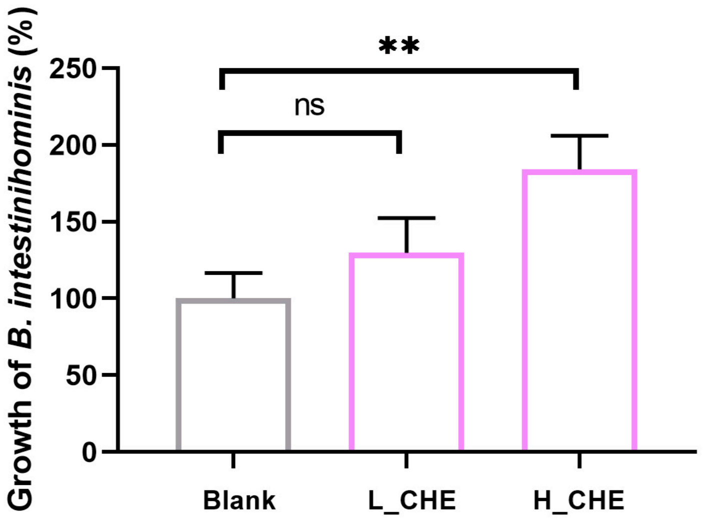

Figure S1. *B. intestinihominis* was treated with CHE or blank control for 48 hours, and bacterial growth was then analyzed by measuring turbidity at 600 nm.

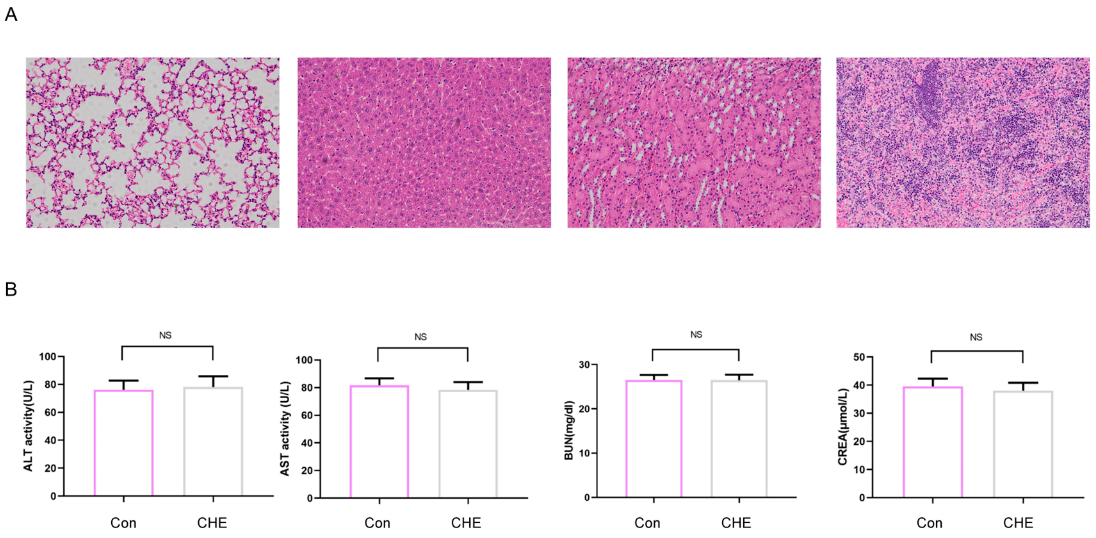

Figure S2. Pathological effects of high-dose CHE treatment on the lung, liver, kidney, and spleen of mice. (A) Pathological effects of high-dose CHE treatment on the lung, liver, kidney, and spleen of mice. (B) Serum ALT, AST, BUN, and CREA level of mouse.

Supplementary Table S1. Antibodies used in this study:

Antibodies	Source	Identifier
Anti- Phospho-STAT3	Cell Signaling Technology	Cat#9134
Anti- STAT3	Cell Signaling Technology	Cat#4904
Anti- Phospho-ERK1/2	Proteintech	Cat#28733-1-AP
Anti- ERK1/2	Proteintech	Cat#11257-1-AP
Anti- Phospho-JNK	Cell Signaling Technology	Cat#4668
Anti- JNK	Proteintech	Cat#51151-1-AP
Anti- Phospho-p38	Proteintech	Cat#28796-1-AP
Anti- p38	Proteintech	Cat#14064-1-AP
Anti-β-actin	Proteintech	Cat#66009-1-Ig

Supplementary Table S2. Primer sequence:

Gene	Primer	Sequence
Nrf2	Forward	CGAGATATACGCAGGAGAGGTAAGA
	Reverse	GCTCGACAATGTTCTCCAGCTT
HO-1	Forward	TGCAGGTGATGCTGACAGAGG
	Reverse	GGGATGAGCTAGTGCTGATCTGG
GCLC	Forward	CAGTCAAGGACCGGCACAAG
	Reverse	CAAGAACATCGCCTCCATTCAG
TNFα	Forward	CCCCTTTATTGTCTACTCCTCA
	Reverse	TCACTGTCCCAGCATCTTGT
IL17α	Forward	ATCCCTCAAAGCTCAGCGTG
	Reverse	TCAGGGTCTTCATTGCGGTG
Cxcl2	Forward	GGCGGTCAAAAAGTTTGCCT
	Reverse	TTCTTCCGTTGAGGGACAGC
Ccl2	Forward	CTCACTGAAGCCAGCTCTCTC
	Reverse	CTGGACCCATTCCTTCTTGGG
Cxcl5	Forward	GAGCTGCGTTGTGTTTGCTT
	Reverse	ACTGGCCGTTCTTTCCACTG
GAPDH	Forward	TCTCCTGCGACTTCAACA
	Reverse	TGGTCCAGGGTTTCTTACT
